# Development of a Wearable Biocueing App (Sense-IT) Among Forensic Psychiatric Outpatients With Aggressive Behavior: Design and Evaluation Study

**DOI:** 10.2196/29267

**Published:** 2021-11-24

**Authors:** Annemieke ter Harmsel, Thimo van der Pol, Lise Swinkels, Anna E Goudriaan, Arne Popma, Matthijs L Noordzij

**Affiliations:** 1 Inforsa, Forensic Mental Health Care Amsterdam Netherlands; 2 Department of Child and Adolescent Psychiatry and Psychology Amsterdam Public Health Research Institute Amsterdam UMC, Vrije Universiteit Amsterdam Amsterdam Netherlands; 3 Department of Research and Quality of Care Arkin Amsterdam Netherlands; 4 Department of Psychiatry Amsterdam Public Health Research Institute Amsterdam UMC, University of Amsterdam Amsterdam Netherlands; 5 Department of Psychology, Health and Technology University of Twente Enschede Netherlands

**Keywords:** biocueing, biosensing, biofeedback, aggression, forensic psychiatry, wearable technology, mobile apps, usability, evaluation, mHealth

## Abstract

**Background:**

The ability to regulate anger is often impaired in forensic psychiatric patients, frequently resulting in aggressive behavior. Although some treatment programs are partially successful in enhancing aggression regulation and reducing recidivism among specific subgroups, generalizable conclusions on the effectiveness of these interventions cannot be drawn to date. In forensic outpatient care, low treatment adherence and a predominant focus on cognitive control in most treatment programs may entail some of the factors impeding treatment. Technology-based interventions may address some of these treatment challenges.

**Objective:**

The aim of this study is to explore whether a new technology-based biocueing intervention, the Sense-IT app, can be a valuable addition to aggression regulation treatment programs in forensic outpatient care. The Sense-IT app, which provides the user with real-time physiological feedback and behavioral support, is developed to strengthen emotional awareness and facilitate real-life practice. In this study, we aim to develop and evaluate an updated version of the Sense-IT app that is suitable for forensic outpatients with aggressive behavior.

**Methods:**

First, we conducted a design study to assess the attitudes of forensic professionals and patients toward biocueing and to collect requirements for a biocueing app for this specific population. On the basis of this information, we developed an updated version of the Sense-IT app. In an evaluation study, 10 forensic outpatients used the app for 2 weeks. The app’s acceptability, usability, and clinical outcomes (aggression, anger, and recognition of bodily signals related to anger) were measured before and after the intervention using both quantitative and qualitative measures.

**Results:**

The design study revealed a cautiously positive attitude toward the use of biocueing as an addition to aggression regulation therapy. The evaluation study among forensic outpatients demonstrated moderate acceptability and adequate usability for the new version of the Sense-IT app. Exploratory analysis revealed a significant decrease in trait aggression postintervention; no significant changes were found in other anger-related clinical outcomes. To further increase acceptability and usability, a stable functioning app with self-adjustable settings, the use of smartwatches with a longer battery life, and the use of the patient’s own smartphone devices were recommended.

**Conclusions:**

This study, which is one of the first attempts to enroll and evaluate the real-life use of a biocueing intervention among forensic outpatients, emphasized the importance of involving both patients and therapists throughout the development and implementation process. In the future, experimental studies, including single-case experimental designs using ecological momentary assessment, should be performed to evaluate the effectiveness of the Sense-IT intervention on clinical outcomes. An open attitude toward new technology, allowing exploration of the potential benefits of the Sense-IT app case-by-case, and training of therapists in using the app are expected to facilitate its integration in therapy.

## Introduction

### Background

Aggression and violent behavior are associated with substantial problems, especially for the victims and the offenders. While victims of violence are at a high risk of developing psychological and behavioral problems such as depression, anxiety, posttraumatic stress, and alcohol abuse [1,2], offenders show increased rates of developing psychiatric disorders, such as psychotic, mood, and substance use disorders [3,4]. In addition, the offenders’ imprisonment often leads to more unfavorable situations for them after release, such as job loss, housing problems, and a lack of social support. Consequently, aggressive behavior is a high burden for professionals working in the mental health care and the judicial system, resulting in inflated costs for both health care and the society in general [5,6]. The impact of aggressive behavior on both individual lives and society in general highlights the importance of early and effective treatment for forensic psychiatric patients with problematic aggressive behavior. Given the current trend of preferring outpatient interventions over residential treatment, the importance of real-life, out-of-session practice of behavioral alternatives [7], and the developments in the use of digital technology in psychological treatment [8], technology-based interventions are of interest to improve and support aggression regulation among forensic (outpatient) populations.

Over the years, several therapeutic interventions have been developed to reduce aggressive behavior and criminal recidivism among forensic in- and outpatients. Most offender treatment programs are based on the principles of aggression replacement training (ART) [9], in which behavioral, affective, and cognitive components are combined to improve anger and aggression regulation. In forensic psychiatry, these cognitive behavioral therapy (CBT) programs are broadly considered as promising rehabilitative treatments for antisocial behavior [10,11]. However, mixed findings have been reported regarding the effectiveness of these treatment programs. A meta-analysis of 14 CBT-informed anger management studies revealed an overall 28% risk reduction in violent recidivism after treatment, with a 56% reduction among those who completed the treatment. In half of the studies, significant differences in violent reoffence were reported, when compared with control conditions [12]. In a systematic review, including 16 ART studies, researchers stated that generalizable conclusions could not be drawn owing to, among other factors, differences in the severity of psychopathology, low methodological quality, and limited follow-up information [13].

The limited effectiveness of current treatment programs is also related to challenges specific to forensic populations. First, because of severe psychopathology, a lack of problem awareness, and motivational difficulties, treatment engagement and adherence are often low. Among forensic populations, the rates of pretreatment drop out and treatment attrition are high. A large meta-analysis revealed an overall treatment attrition rate of 27.1% across all offender programs, with a rate of 37.8% for domestic violence offenders [14]. Research results indicate that noncompletion of treatment is associated with lower reductions in general and violent recidivism [12]. Furthermore, homework and registration assignments—an important part of all CBT programs—are often not completed in forensic outpatient populations. This negatively affects the transfer of psychological interventions from the therapist room to daily practice [15]. Second, the focus on cognitive control over emotional processes—a core element of most current treatment programs—may not fit the capacities of the patients. Forensic psychiatric patients often lack insight into their emotions [16] and have difficulty in observing and interpreting physiological signs of increased inner tension, such as accelerating heartbeat, sweating, or trembling. This limited capability to timely detect (particularly slowly) rising arousal levels increases the chance of *suddenly* occurring aggressive outbursts. Furthermore, cognitive control processes can be overruled by impulsive aggressive behavior in the case of high physiological arousal [17]. Therefore, in line with previous literature [18], therapy should focus more on strengthening awareness of bodily sensations associated with anger, before moving to the enhancement of cognitive control and deliberate responses in anger-provoking situations.

New technological applications may help address some of these treatment challenges. Technology-based interventions, such as mobile biofeedback apps [19], serious gaming [20], and virtual reality therapy [21] have the potential to increase adherence to treatment by engaging patients and by increasing maintenance during out-of-session activities [22]. Considering their lack of emotional awareness, interventions that provide the patients with information about their physiological state in real-life situations may help signal heightened arousal in response to emotional events and support adequate self-regulation [23,24]. In this study, we aim to explore the potential of a new biocueing intervention, which signals *at risk* levels of arousal in everyday life, as an addition to current aggression regulation therapy.

Biocueing can be seen as a specific, personalized type of biofeedback [25]. In the process of biofeedback, instruments monitor physiological parameters (eg, heart rate, skin conductance, and respiration), transform these measurements into auditory or visual signals, and present these signals to the user directly [26]. During a traditional, nonwearable biofeedback paradigm consisting of multiple on-site sessions, patients are trained to regulate their physiological reactions by consciously alternating their responses to the given feedback. Rapid developments in noninvasive, wearable technology (eg, breast bands, wrist sensors, smart fibers, and interactive textiles) have opened opportunities for biocueing, which combines real-time measurement in everyday life and just-in-time behavioral support [27-29].

Traditional biofeedback has proven effective for patients with different psychopathology; however, most studies have been conducted among patients with internalizing problems [24]. Biocueing is relatively new but might be particularly useful for patients who lack insight into the physiological signals that precede dysregulated behavior in everyday life, such as binge-eating episodes [30] or self-injurious behavior [31]. Focusing on aggression, several pilot studies have provided the first evidence that physiological information can be used as a predictor of aggressive behavior, for example, among youth with autism spectrum disorder [32], patients with intellectual disabilities [33], and forensic patients [34]. Therefore, biocueing might be a helpful tool to increase awareness of high-risk situations and to support patients in practicing behavioral skills that prevent their escalation into aggressive incidents [35]. However, there is a gap between these study results and the actual deployment of mobile health (mHealth) interventions, such as a biocueing app for wearables, in forensic clinical practice. To bridge this gap, consideration of the needs of the intended users, as well as usability evaluation in the user’s natural environment, is required [36]. The involvement of end users throughout the design process, the core principle of user-centered design, is therefore highly recommended for the development of useful and effective mHealth interventions [37].

### Study Aims

Given the potential of biocueing in dealing with the challenges in forensic (outpatient) treatment programs and using the principles of user-centered design, we first explored the attitudes of forensic professionals and patients toward this new intervention as an addition to aggression regulation therapy. In addition, we collected requirements to develop an updated version of the Sense-IT biocueing app [38] for use in a forensic outpatient population. Finally, we investigated the acceptability and usability of the revised Sense-IT app and explored changes related to aggression, anger, and interoceptive awareness in a 2-week evaluation study among 10 forensic outpatients with aggressive behavior.

## Methods

### Overview

**Figure 1:**
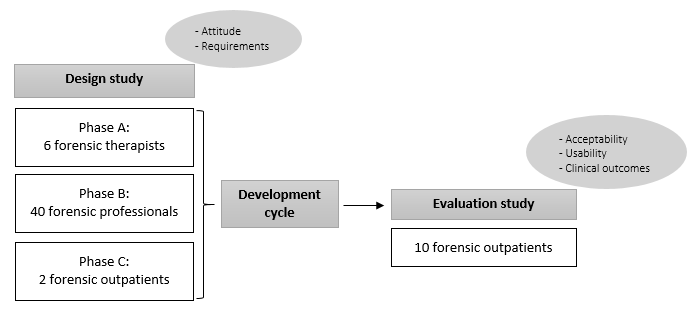
Study design: phases, participants, and concepts.

First, we conducted a design study to explore the attitudes toward and specific requirements for a biocueing intervention in a forensic sample. With this information, an updated version of the Sense-IT app was developed. Then, we studied the app’s acceptability and usability and its preliminary effects on clinical outcomes in a 2-week evaluation study. The structure of the study has been shown in Figure 1.

### Design Study

From October 2016 to March 2017, 6 forensic therapists, 40 forensic professionals (ie, therapists, psychiatric nurses, social workers, and probation officers), and 2 forensic outpatients participated in our study. We informed all the participants of the study’s content and the voluntary basis for participation. We recruited the forensic therapists by email and the forensic professionals through in-person engagement at a forensic congress. The forensic outpatients were approached after consultation with their therapists, and their signed informed consent was obtained. The forensic therapists and outpatients tested a precursor version of the Sense-IT app for 3 to 7 days, respectively, using a research-owned smartphone and smartwatch. The Sense-IT system provides a visual display of physiological arousal by measuring heart rate, notifies the user of level changes, and delivers a default message when the user’s physiological arousal is significantly elevated (SD >2) than their personal baseline. In this design study, the baseline measurement consisted of 300 reliable heart rate measures, with 20 seconds between each measurement. During the baseline measurement, participants could behave normally, without any restrictions. The forensic professionals responded to a short paper-and-pencil survey to increase their knowledge regarding their attitude toward biocueing. None of the participants received any financial compensation for this study.

Given the outcomes of this study, the forensic professionals’ attitudes toward biocueing could be considered open and cautiously positive. The participants mentioned awareness of bodily signals accompanying anger (9/40, 22%) and insight into increasing arousal levels, especially in high-risk situations (15/40, 37%), as the most valuable additions to aggression regulation therapy. Furthermore, biocueing was seen as a promising way to open therapeutic conversations about aggressive behavior. According to most forensic therapists (4/6, 67%), the usability of this precursor version of the Sense-IT app was insufficient. The limited battery life of the watch, the sudden watch face changes, the synchronization problems, the questionable reliability of heart rate measurements, the feedback method (too soft and too slow), and the mandatory use of a research phone were listed as items for improvement. Therapists were most satisfied with the visualization of arousal on the smartwatch and the warning function for heightened arousal. Of the 2 forensic outpatients, only one used the app for the entire week. This participant recommended audio-recording because he had difficulty typing notes. He reported an increased awareness of arousal, increased control over his aggressive behavior by the initiation of self-calming strategies, and distraction from inner tension by using the Sense-IT system. The other participant quit the study early because of technological shortcomings in this version of Sense-IT. This participant was frustrated with the app interrupting measurement when the watch face was accidently touched. Given the insufficient usability scores and the increased irritability reported by one of the patients, we initiated a development cycle to resolve these technological shortcomings before further rolling out the app among forensic outpatients.

### Development Cycle

Technological stabilization was the most important aim of this development cycle. The recommended improvements were implemented and intermediately tested by the researchers and app developers in 5 iteration rounds between 2017 and 2018. The activation of the Sense-IT app on the smartphone and smartwatch was synchronized and automated to prevent synchronization problems. Furthermore, the connection between the smartwatch and smartphone was made visible on the main screen. A clear on-off slider was incorporated into the main screen. Continuous visualization of the data on the watch face was optimized. Finally, the user was allowed to define during which activity profiles (eg, driving, cycling, and running) the operation of the Sense-IT should be paused. A description of the revised Sense-IT app has been provided in the next section.

### Evaluation Study

#### Participants

**Figure 2:**
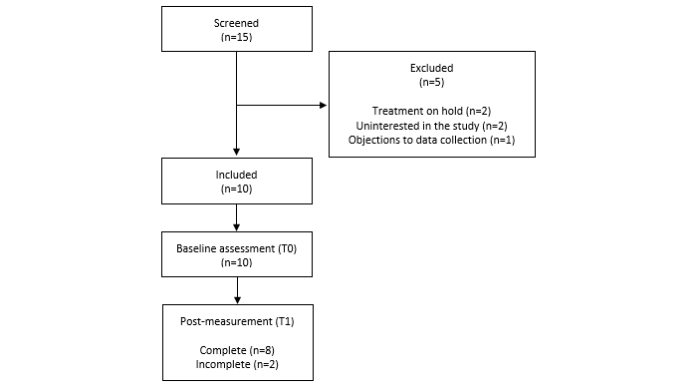
Flow chart of recruitment and participation in the evaluation study.

We recruited forensic outpatients receiving aggression regulation therapy at Inforsa for participation from November 2018 to July 2019. Inforsa is a forensic mental health care facility that specializes in the treatment of patients with disruptive and criminal behavior. A research associate screened the potential participants for eligibility, in consultation with the patient’s therapist. The eligibility criteria included (1) a proven lack of anger management skills, indicated by either a recently committed violent crime and/or a high risk of committing one, (2) assignment to individual outpatient aggression regulation treatment after multidisciplinary consultation, (3) basic understanding of mobile apps, and (4) aged ≥16 years. The exclusion criteria included (1) acute manic or psychotic symptoms, (2) current high risk of suicide, (3) severe addiction problems or other severe conditions requiring immediate intervention or hospitalization, and (4) insufficient understanding of the Dutch language. The first 3 exclusion criteria were assessed using cut-off scores on the corresponding items in the Health of the Nation Outcome Scales [39]. A total of 10 forensic outpatients were enrolled in the study. An outline of the recruitment and participation flow is displayed in Figure 2.

#### Procedure

This study was approved by the Medical Ethical Committee of Amsterdam University Medical Centre, the Netherlands (NL63911.029.17). This study was also registered in the Netherlands Trial Register (NL8206). If a patient was eligible and interested in the research project, study participation was offered during a face-to-face appointment with the therapist and the patient. The research associate provided the patient with a brief oral description and full written information on the study. The voluntary nature and the absence of any negative consequences for the patient’s refusal to participate were emphasized. If the patient was interested, the next appointment was planned after at least 7 days, providing enough time for consideration. In this appointment, we obtained the informed consent of the participants, and they filled out self-reported questionnaires. The baseline measurement (T0) lasted approximately 45 minutes. After completion of the assessment, participants were provided with a smartwatch and a mobile phone with the Sense-IT app. The participants were shown how to use the devices and were given tips on charging and using the system safely. They also received a user manual. The participants used the devices independently during the following 2 weeks. They were encouraged to call the research associates if any problems occurred. After the 2-week intervention period, another 45-minute assessment (T1) was planned. We used the same measurements as at T0, supplemented with qualitative interviews and quantitative usability measures.

#### Materials

##### Demographics

We collected demographic and clinical information at T0 using a 23-item self-developed questionnaire. The variables assessed included gender, ethnicity, judicial history, care history, education, family background, and social situation.

##### Mobile Phones and Smartwatches

The participants received both a smartwatch and a mobile phone. In this evaluation study, we used the Ticwatch E (Mobvoi, Ltd), a new smartwatch that had good reviews on reliability and cost-effectiveness and had a longer battery life than the smartwatches we used in the design study (Moto 360 2nd Gen; Lenovo Group, Ltd). The smartwatches were equipped with a photoplethysmography (PPG) sensor, by which the blood volume pulse can be measured and the heart rate can be derived. Connection with the mobile phone, the Moto C Plus (with Android 8.0 operating system; Google, LLC), was established via Bluetooth. We provided the participants with research-owned mobile phones to maintain control over the app settings and to account for secure data extraction.

##### Sense-IT App

**Figure 3:**
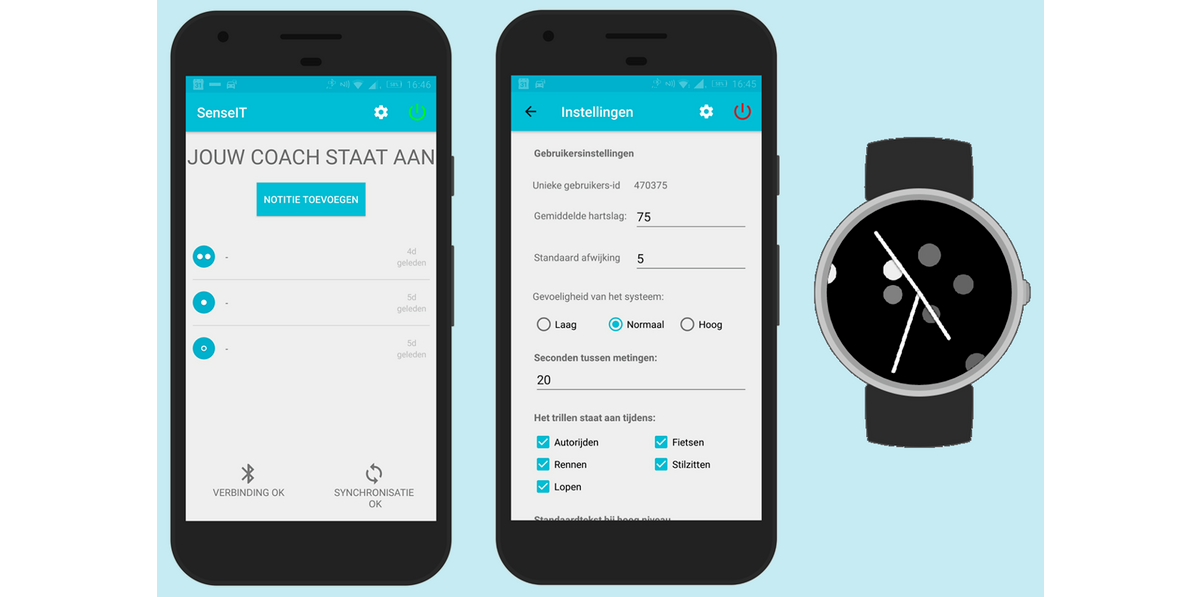
Screenshots of the Sense-IT app (version 2.13): main screen with measurements, settings screen, and the watch face.

The newly developed version of the Sense-IT app, version 2.13, was preinstalled on all smartwatches and mobile phones before distribution. The Sense-IT app was originally developed by researchers at the University of Twente in co-operation with Scelta, an expert center for psychiatric patients with personality disorders [38,40]. The Sense-IT system reads the physiological data measured by the photoplethysmography (PPG) sensor and stores the data in a local database on the smartphone itself. The built-in algorithm compares the current heart rate of a user with their mean heart rate at baseline and calculates a level between −3 and 5 using the SD of the baseline measurement. In this study, the determination of the user’s baseline was started at the end of the T0 measurement and lasted until the PPG sensor received 200 reliable heart rate measures, with 20 seconds between each measurement. During baseline measurement, participants could behave as they normally would, except for any intense physical activity. After this measurement, the level of their current heart rate was visually displayed on the smartwatch and was changed when the heart rate decreased or increased by ≥1 SD. For this study, notifying vibrations were sent to the users at every change in their physiological level. The Sense-IT app detects (physical) activity categories using the accelerometer and Google activity recognition algorithms, allowing the user to receive notifications for certain activity profiles (eg, sitting still and driving a car) only. From the user interface on the smartphone, users can turn the app on and off, opening a timeline of all the measurement events and level changes detected by the system. Users can add notifications to events in the timeline and report their subjective level of arousal, which might be particularly useful when the user is notified of level changes. Users can also define a personalized message that is displayed when their physiological arousal exceeds a predefined level. In this study, we used a default message (ie, “your heart rate is higher than average”), which was only displayed at levels 3, 4, and 5 (SD>2) above baseline. The app’s user interface also presents information about the connection and synchronization status, as well as a settings page protected by a password to prevent unwarranted changes. Screenshots of the Sense-IT app are displayed in Figure 3.

##### Acceptability

In a semistructured qualitative interview developed by the study team, the participants’ attitude toward technological interventions and their perceived proficiency in using new technology were assessed using a 5- and 10-point Likert scale, respectively. Closed-ended and open-ended questions assessed whether the participants would use the Sense-IT app in the future and whether they expected others to do so. The total number of heart rate measurements, measured every 20 seconds by the PPG sensor of the smartwatch, was used as an indicator of the actual use of the Sense-IT system. The damage, loss, and theft of the devices were recorded.

##### Usability

We administered the System Usability Scale (SUS), a short, commonly used questionnaire for quick and reliable assessment of product usability [41], at T1. The SUS consists of 10 statements that can be scored on a 5-point Likert scale, ranging from 1 (*totally disagree)* to 5 (*totally agree*). The SUS yields an overall score between 0 and 100, with higher scores indicating better usability. In the original study, 68 was used as a cut-off score. According to more recent research [42], a product is *acceptable* with scores above 70; better products score in the high 70s to upper 80s and superior products score above 90. Products with scores lower than 70 should be considered as candidates for improvement.

Furthermore, we evaluated usability qualitatively by using semistructured interviews. The interview included Likert scale questions about the attractiveness of the devices, the ease-of-use of the app, the clarity of watch faces, and the evaluation of feedback notifications. Open questions assessed the advantages and disadvantages of Sense-IT and any recommendations for its further improvement.

##### Aggression

We assessed for aggressive behavior using the Dutch version [43] of the Aggression Questionnaire-Short Form (AQ-SF) [44]. The AQ-SF is a self-report questionnaire, in which participants respond to 12 statements regarding aggression on a 5-point Likert scale, ranging from 1 (*strongly disagree*) to 5 (*strongly agree*). The AQ-SF distinguishes 4 subscales: physical aggression, verbal aggression, anger, and hostility. The internal consistency coefficients for the total score of the Dutch AQ-SF ranged between 0.72 and 0.88 in a forensic population. Significant test-retest correlations after 4 weeks were found for the AQ-SF total and subscale scores, except for the physical aggression subscale. The AQ-SF was administered at T0 and T1.

##### Anger

We assessed anger and its subcomponents using the Dutch version [45] of the State-Trait Anger Expression Inventory-2 (STAXI-2) [46]. The STAXI-2 is a 57-item self-report questionnaire, in which items are coded on a 4-point Likert scale ranging from 1 (*almost never*) to 4 (*almost always*). The questionnaire consists of 3 main scales: state anger, trait anger, and anger expression and anger control. The internal consistency, assessed in an inmate sample, was considered good, with Cronbach α ranging from .79 to .88. For the original version of the STAXI, the test-retest coefficients were acceptable, except for the State Anger scale. We administered the full STAXI-2 at T0; at T1, we administered only the state and trait anger scales because of time constraints.

##### Bodily Sensations Related to Anger

We measured bodily sensations related to anger in interpersonal situations using the Dutch version of a recently developed self-reported questionnaire called the Anger Bodily Sensations Questionnaire (ABSQ) [47]. In this 18-item self-report questionnaire, participants can rate their experience of physiological responses during anger-provoking interpersonal situations on a Likert scale, ranging from 1 (*not at all*) to 5 (*very much*). The internal consistency (Cronbach α) for the total score was .93 in an offender population for this study. The total score had good 1-week test-retest reliability within the offender sample. The ABSQ was administered at T0 and T1.

#### Data Analysis

We analyzed the quantitative data using SPSS (version 25, IBM Corp). Although this evaluation study was not intended for inferential statistics, we exploratively compared the pre-post scores, after checking the normality assumptions, with the nonparametric equivalent of the paired *t* test, the Wilcoxon Matched Pairs test. We analyzed the qualitative data using Microsoft Word and Excel. Textual responses were first inspected for theme analysis, then coded into categories and described as relative results. Categorical responses were described as relative results.

## Results

### Demographics

Of the 10 forensic outpatients participating in this study, the majority (9/10, 90%) were male, in line with the usual male-female distribution in forensic populations. Although all participants were born in the Netherlands, out of 10 participants, 6 (60%) had parents originating from another country. Furthermore, 60% (6/10) reported problems such as domestic violence, substance abuse, or psychological problems in the families they grew up in. Most of the participants were referred to Inforsa for mandatory treatment as part of a conditional sentence and had been convicted multiple times in the past. The descriptive characteristics of the participants have been summarized in Table 1.

**Table 1:** Summary of demographic characteristics of the participants^a^ (N=10).

Demographic characteristics				Values
Age (years), mean (SD)				34.90 (13.29)
**Gender, n (%)**				
	Male			9 (90)
	Female			1 (10)
**Educational background, n (%)**				
		Primary school	2 (20)	
		Secondary school	2 (20)	
		Secondary vocational education	6 (60)	
**Living conditions, n (%)**				
		Private home	4 (40)	
		Assisted living facility	4 (40)	
		Social care	2 (20)	
**Index offense, n (%)**				
		Violent crime	5 (50)	
		Gun crime	1 (10)	
		No index offense	4 (10)	
Multiple convictions, n (%)				8 (80)
Mandatory treatment, n (%)				6 (60)
Previous ART^b^ treatment, n (%)				5 (50)

^a^Number of participants measured at T0.

^b^ART: aggression replacement training.

### Acceptability

The participants were provided with the Sense-IT system for 2 weeks. All participants returned these research-owned smartwatches and mobile phones. Although we noticed some superficial user damage and had to buy new charging cables, none of the devices had to be replaced because of damage, loss, or theft. Half of the participants (5/10, 50%) fully agreed with the statement that they liked to use technology or technological gadgets, a substantial number (4/10, 40%) claimed a neutral position and 1 participant (1/10, 10%) disagreed with the statement. On a 10-point Likert scale, the participants rated themselves as proficient in using new technologies (mean 7.1, SD 2.5 participants). At T1, it appeared that one participant had not used the Sense-IT app at all; the T1-responses of this participant were therefore excluded from further analysis. Among the other participants, the total amount of heart rate measurements (in hours) was used to indicate actual app use. As some participants showed very large numbers of measurements per day, we corrected the data for very low heart rates (<50 beats per minute) and measurements during nighttime (from midnight until 6 AM). The corrected actual app usage strongly differed between participants (mean 62.19, SD 38.63 hours; range 3.49-127.02 hours). Higher heart rate measurements were found among older participants, *r*_8_=.768, *P=*.016. No significant correlations were found among the attitude toward new technology, the perceived proficiency, and the indicator of actual app use. Furthermore, out of 9 participants at T1, 6 (67%) reported that they would like to use the Sense-IT app in the future. All the participants expected others to use the app in the future.

### Usability

Participants who said they would not use the Sense-IT app in the future reported that the app had no added value for them because, for example, they did not regard themselves as aggressive or they already claimed to know their personal precursors for aggressive behavior. One participant therefore recommended the addition of the Sense-IT app in the early phases of treatment. The participants who would use the app in the future listed several conditions that could be considered as recommendations to further improve the Sense-IT app. The average score on the SUS for the group was above the cut-off value (mean 73.1, SD 16.2). Significantly higher system usability scores were reported by participants with a more positive attitude toward new technology, *r*_9_=0.857, *P*=.002; no significant correlations were found between the usability scores and perceived proficiency in using new technology. Most participants (8/9, 89%) did not report difficulty using the Sense-IT app on the smartphone. The watch faces on the smartwatch were reported to be clearly visible by the participants. The design of the Sense-IT app on the smartphone was considered neutral by half of the participants and (quite to very) attractive by the other half; some participants reported that they would like a more colorful design. Considering the messages shown when the physiological values exceeded a predefined level, out of 9 participants, 6 (67%) said they would like to use a default text message; the other participants (3/9, 33%) preferred a personalized message. The number of notifying vibrations (delivered at every level change) was considered too large by 56% (5/9) of the participants and was therefore most often mentioned as a point of improvement. Furthermore, improved accuracy, longer smartwatch battery life, and the possibility of using the Sense-IT app on their own smartphones were recommended.

### Clinical Outcomes

We performed an exploratory analysis on the clinical outcome measures. Given the small sample size, the Shapiro-Wilk test was used to evaluate for normality of the data. As expected, the normality assumption was not met for several subscales of the measures used. Therefore, the Wilcoxon Matched Pairs test was used. No significant changes were found on the AQ-SF and ABSQ. Between baseline (median 2.35) and postmeasurement (median 1.90), trait anger measured with the STAXI-2 decreased significantly (*Z*=−2388; *P*=.017). Explorative visual analysis of the data showed that the scores of most of the participants (7/9, 78%) decreased. None of the participants showed elevated scores at postmeasurement compared with baseline. No significant correlations were found between this trait anger decrease and other variables such as attitude toward new technology, perceived proficiency in using new technology, and usability of the Sense-IT app. All clinical outcomes are shown in Table 2.

**Table 2:** Summary of the clinical outcomes.

Clinical outcome scores			Time points		
			T0 (n=10), mean (SD)		T1 (n=9), mean (SD)
**Aggression (AQ-SF^a^)**			2.36 (0.78)		2.41 (0.85)
	Physical aggression	2.23 (1.21)		2.23 (1.32)	
	Verbal aggression	2.23 (0.96)		2.63 (0.78)	
	Anger	2.87 (1.00)		2.73 (1.31)	
	Hostility	2.10 (1.03)		2.03 (1.08)	
**Anger (STAXI-2^b^)**			1.91 (0.35)		—^c^
	State anger	1.05 (0.13)		1.02 (0.05)	
	Trait anger	2.21 (0.65)		1.98 (0.72)	
	Anger expression and control	2.22 (0.48)		—	
Bodily sensations related to anger (ABSQ^d^)			2.34 (0.93)		2.49 (0.95)

^a^AQ-SF: Aggression Questionnaire-Short Form.

^b^STAXI-2: State-Trait Anger Expression Inventory-2.

^c^Missing data.

^d^ABSQ: Anger Bodily Sensations Questionnaire.

## Discussion

### Principal Findings

To our knowledge, this study is one of the first attempts to enroll and evaluate a smartwatch-based biocueing intervention in a forensic outpatient population with aggression regulation difficulties [25]. Our study revealed a cautiously positive attitude toward the use of biocueing as an addition to regular therapy. Requirements for improvement were processed in a development cycle, resulting in an updated version of the Sense-IT app. The results of our 2-week evaluation study showed adequate usability scores, although the actual use of the app and its expected future use did not entirely match these outcomes. Furthermore, a significant decrease in trait anger was observed postintervention. Valuable recommendations were obtained for further improvement in Sense-IT. Considering the aim of our study, we were able to collect relevant information for the further development and enrollment of technology-based interventions as adjuncts to treatment, even in populations with lower treatment adherence.

In accordance with the principles of user-centered design for mHealth applications [37], which had also been applied in the earlier development phases of the Sense-IT app [38,40], we involved patients, therapists, and other forensic professionals in the development process of Sense-IT. The recommendations collected in the design study were mainly related to technological issues impeding the ease-of-use, such as synchronization problems and limitations in the battery life of the smartwatch. As the usability of the precursor version of the Sense-IT turned out to be inadequate for extensive testing among forensic outpatients, its further development was initiated. To generate more input for this development cycle, the professionals’ attitudes toward the use of a biocueing app for this particular group were more thoroughly assessed. Our design study indicated that forensic professionals had an open and positive attitude toward biocueing, recognizing the potential disadvantages or risks. This is important because openness toward new treatment possibilities and a belief that the intervention might be beneficial to patients are, among others, considered as facilitators of the use of mHealth technology [48,49].

Patients’ adoption of mHealth applications is also known to be a result of several factors, such as perceived usefulness and ease-of-use, influencing their individual attitude and behavioral intention, mediated by age [50]. Other results suggest that the perceived mobile technology identity, related information technology experience, and self-efficacy are associated with higher adoption rates [51]. In our evaluation study, the users’ perceived proficiency was not associated with the actual use and the perceived usability of Sense-IT. Actual use seemed higher among older participants, contrary to the mediating influence of age, as mentioned in other studies. However, the higher usability scores being reported by patients with a more positive attitude toward new technology was consistent with these earlier findings. Furthermore, we encountered some skepticism when we first presented our idea to investigate a biocueing intervention among forensic outpatients by providing them with a smartwatch and smartphone. Contrary to expectations, none of the devices had to be replaced because of loss, damage, or theft, supporting a recent study among homeless youth [52]. The total amount of heart rate measurements, used as an indicator for the actual app usage, varied widely among the participants. Most participants used the Sense-IT quite often, but a few participants showed very low adherence to the app. This limited adherence to technology-based interventions is a common issue in eHealth and mHealth studies, even in samples that are more open to treatment. Many users of self-help applications show inconsistent use patterns [53], do not continue their use after completion of one exercise or module [54], or stop using a health app after 2 weeks, especially when their preferences and goals are not met [55]. Given the answers regarding the future use of the Sense-IT app, actual usage of the app might have decreased even more if the duration of our study had been extended to more than 2 weeks. Nevertheless, considering the motivational problems and the high dropout rates in forensic populations, outpatients who often fail to practice outside the treatment setting would be encouraged to reflect on their behavior in real life.

Furthermore, the Sense-IT app received acceptable system usability scores, which means that the ease-of-use was considered *good enough* for further development and can be rolled out in this population. The participants provided valuable recommendations for a new development cycle. The number of notifying vibrations, which were considered disturbing by most of the participants, was most often mentioned as a point for improvement. For some patients, this feature of the app led to increased irritability, a possibility that has already been mentioned in previous literature [56-58]. In the future, this drawback can easily be remedied by adjusting the levels at which notifying vibrations are provided and by allowing users to customize the settings themselves. Furthermore, our study revealed that some participants had difficulty coming up with a personalized message, which could be shown when their physiological values were elevated. Therefore, their preference for the default text message might have been a choice out of convenience. Further integration of the Sense-IT app in therapy may help to overcome this difficulty. At the design level, desired improvements, such as the addition of multiple colors, were mostly mentioned by younger participants. Furthermore, several participants would have liked to use Sense-IT on their own mobile phones. For clinical use, outside a research context with privacy constraints, this would certainly be possible and might facilitate the acceptability and usability of the app. Finally, technological hardware improvements, such as the extended battery life of the smartwatch, could further enhance adherence to Sense-IT.

To investigate whether the 2 weeks of using the Sense-IT biocueing app were associated with clinically relevant changes, we performed exploratory analysis on the pre and post measurement outcomes of aggression, anger, and anger bodily sensations. In line with the literature indicating the difficulty of changing aggressive behavior [13] and given the small sample size and short intervention period of our study, no significant changes were found in the overall scores. Considering the concepts of state and trait anger, in this study, change might theoretically occur on state anger. Interestingly, trait anger diminished significantly between pre- and postintervention measurements. No change was found in the state anger. However, this might have been affected by the fact that state anger was only measured at 2 specific moments in time, when the participants were generally in a resting state. Regarding the change in trait anger, social desirability might offer a partial explanation for this finding. In a study among forensic inpatients, participants with high scores on impression management reported significantly lower levels of trait anger [59]. To explore whether this finding reflects a true change in the frequency of experiencing angry feelings should therefore be more thoroughly assessed within a longitudinal research design, using assessment methods that are more sensitive to minor changes and less susceptible to social desirability.

### Limitations

Our study had several limitations. First, the number of forensic outpatients participating in the design and evaluation study was low. In line with the early phase of biocueing research and the often-encountered difficulties in studies among forensic patients, this may have affected the findings of our study. Second, we used a small subset of questionnaires in our evaluation study, focusing on anger and aggression. Therefore, we might have overlooked other relevant changes, for example, in emotion regulation in general. In addition, we did not use the full STAXI-2 at T1, which should have been preferred to prevent data loss. Furthermore, the questionnaires we used might have been susceptible to social desirability. In any case, the most used questionnaires to assess aggressive behavior are not designed to detect small changes over short periods. In this regard, the short duration of the intervention and the absence of a follow-up measurement complicate the interpretation of our findings. Third, because we used a pretest-posttest design without a control group, we were not able to disentangle the impact of the use of the Sense-IT biocueing intervention from aggression regulation therapy and from the distribution of mobile phones and smartwatches, which might be considered an intervention itself. Fourth, in some cases, Sense-IT seemed to be measuring data while not being actually worn by the participants. This complicated the interpretation of the actual usage of the device. Fifth, as Sense-IT could not be used on the participants’ own phones, owing to privacy constraints in research, it might have had a restrictive effect on adherence. Sixth, the explanation and handing out of the devices was done by research associates in the absence of their therapists. In addition, both patients and therapists were not able to adjust the settings during the study. These restrictions, associated with the research design, might have limited the adoption of the Sense-IT app by therapists, an important driver for the integration of mHealth interventions in clinical practice [49].

### Implications for Future Research and Practice

**Figure 4:**
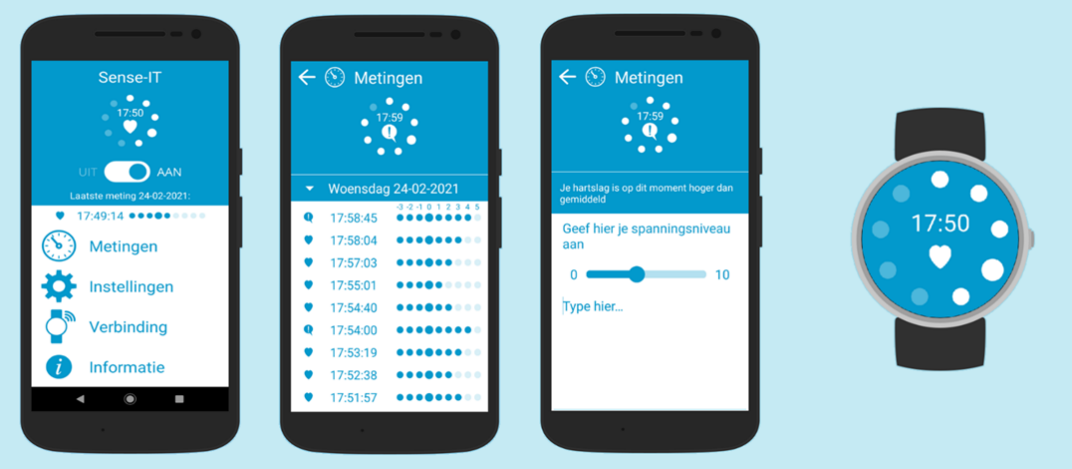
Screenshots of the Sense-IT app after further development (version 2.57): main screen, measurement screen, note screen, and the watch face.

This development and usability study has several key implications for future research. First, it emphasized the importance of the involvement of both patients and therapists in the development of effective mHealth interventions [35]. Second, the evaluation study yielded new recommendations for the improvement of the Sense-IT app. At the technological level, stabilization of the app should remain a critical area for improvement. The number of notifying vibrations and the levels at which SMS text messages are sent should be adjustable to the wishes and needs of patients. In addition, the ease-of-use of recording subjective arousal levels should be enhanced. At the design level, the clarity of the main screen should be improved, and the measurement screen should be updated with a semigraphical representation. To further enhance the attractiveness of Sense-IT, some patients would like to choose different background colors. Meanwhile, the recommendations were processed in a new development cycle, resulting in an updated version of Sense-IT, as shown in Figure 4. Changes in app settings and the use of new smartwatches should be considered to improve the battery life and, thereby, usability. Third, experimental studies should be performed to evaluate the effectiveness of the Sense-IT intervention on clinical outcomes. Single-case experimental designs should be considered as well because these types of designs might be better able to detect minor changes over short periods by, for example, using ecological momentary assessment [60,61]. Laboratory tasks could also be considered to gain a broader insight into the response to biocueing. Fourth, the integration of therapy should be enhanced. To experience the potential benefits of this biocueing intervention, therapists should first be trained in working with Sense-IT and familiarizing themselves with the app. Furthermore, both therapists and patients should be able to adjust the settings to explore the optimal level of sensitivity of the system. Fifth, the Sense-IT app should preferably be added to treatment in the early phases of aggression regulation therapy, focusing on the recognition of anger bodily signals. The use of new technological interventions might have a positive influence on treatment motivation [21], especially among patients with a positive attitude toward new technology.

### Conclusions

This study revealed a cautiously positive attitude toward the use of biocueing as an addition to regular aggression regulation therapy in forensic psychiatry. In the evaluation study among forensic outpatients, the revised version of the Sense-IT app demonstrated moderate acceptability and adequate usability. Furthermore, a significant decrease in trait anger was found postintervention, which should be further explored in future research using appropriate research designs. Valuable recommendations for improvement of Sense-IT, both at the technological and design levels, were obtained in this study. For patients and therapists, a stable functioning app, with few synchronization disruptions and a self-adjustable number of notifications, seemed most important. Implementing new smartwatches with a longer battery life and using Sense-IT on the user’s own smartphone are expected to increase adherence in the future. Considering the actual use of Sense-IT, the app seemed to have facilitated out-of-session practice and might therefore represent an alternative for more traditional paper-and-pencil registration assignments. The extent to which users actually reflect on their behavior and whether they feel supported by Sense-IT to practice behavioral alternatives needs further examination. Furthermore, this study provided some evidence that the deployment of Sense-IT is most useful in the first phase of aggression regulation therapy. Finally, to enhance the app’s integration in treatment, therapists should be trained in the use of the app to facilitate exploration of the potential benefits of these kinds of new mHealth interventions with their patients.

## Abbreviations

ABSQ: Anger Bodily Sensations Questionnaire
AQ-SF: Aggression Questionnaire-Short Form
ART: aggression replacement training
CBT: Cognitive Behavioral Therapy
mHealth: mobile health
PPG: photoplethysmography
STAXI-2: State-Trait Anger Expression Inventory-2
SUS: System Usability Scale
